# The invasive cactus *Opuntia stricta* creates fertility islands in African savannas and benefits from those created by native trees

**DOI:** 10.1038/s41598-021-99857-x

**Published:** 2021-10-21

**Authors:** Ana Novoa, Llewellyn C. Foxcroft, Jan-Hendrik Keet, Petr Pyšek, Johannes J. Le Roux

**Affiliations:** 1 Department of Invasion Ecology, Institute of Botany, Czech Academy of Sciences, 252 43 Průhonice, Czech Republic; 2 Department of Botany and Zoology, Centre for Invasion Biology, Stellenbosch University, Stellenbosch, South Africa; 3 Invasive Species Programme, South African National Biodiversity Institute, Kirstenbosch Research Centre, Claremont, South Africa; 4 Scientific Services, South African National Parks, P/Bag X402, Skukuza, 1350 South Africa; 5 Department of Botany and Zoology, Stellenbosch University, Stellenbosch, South Africa; 6 Department of Ecology, Faculty of Science, Charles University, Viničná 7, 128 44 Prague, Czech Republic; 7 Department of Biological Sciences, Macquarie University, Sydney, NSW 2109 Australia

**Keywords:** Invasive species, Plant ecology

## Abstract

The patchy distribution of trees typical of savannas often results in a discontinuous distribution of water, nutrient resources, and microbial communities in soil, commonly referred to as “islands of fertility”. We assessed how this phenomenon may affect the establishment and impact of invasive plants, using the invasion of *Opuntia stricta* in South Africa’s Kruger National Park as case study. We established uninvaded and *O. stricta*-invaded plots under the most common woody tree species in the study area (*Vachellia nilotica* subsp. *kraussiana* and *Spirostachys africana*) and in open patches with no tree cover. We then compared soil characteristics, diversity and composition of the soil bacterial communities, and germination performance of *O. stricta* and native trees between soils collected in each of the established plots. We found that the presence of native trees and invasive *O. stricta* increases soil water content and nutrients, and the abundance and diversity of bacterial communities, and alters soil bacterial composition. Moreover, the percentage and speed of germination of *O. stricta* were higher in soils conditioned by native trees compared to soils collected from open patches. Finally, while *S. africana* and *V. nilotica* trees appear to germinate equally well in invaded and uninvaded soils, *O. stricta* had lower and slower germination in invaded soils, suggesting the potential release of phytochemicals by *O. stricta* to avoid intraspecific competition. These results suggest that the presence of any tree or shrub in savanna ecosystems, regardless of origin (i.e. native or alien), can create favourable conditions for the establishment and growth of other plants.

## Introduction

The past few centuries have seen humans increasingly move plant species around the globe^1,2^. Some of these species have become invasive and pose significant threats to biodiversity by altering the composition and function of invaded ecosystems^3–5^. Despite achievements towards generalization and formulating regional and global strategies to deal with invasive species, it remains important to understand which factors are driving the success and impact of particular invasions in order to prioritize management actions and mitigate the impacts of invasive plants. This is especially urgent in protected areas^6,7^. However, this is no easy task, since invasion success and invader impact are often highly context-dependent, and can differ among taxa, the pathways through which species are introduced or disseminated, or the characteristics of recipient ecosystems (e.g., habitat type, altitude, nutrient availability, abundance of mutualists or natural enemies, disturbance frequency or socioeconomic characteristics and processes)^8,9^.

Savannas constitute the dominant vegetation type in Africa, covering about 60% of the continent^10^. Until recently, African savannas were thought to be less invaded by alien plants compared to other ecosystems^11^. Several reasons have been proposed for this, such as low rate of alien plant introductions in some regions, the adaptation of African savannas to fire (which might prevent the establishment of many alien plants), or the resistance of native plants to disturbance^12^. However, this trend is changing: in the last decades, alien plants (e.g., eucalypts, parthenium weed, or cacti) have been increasingly introduced, becoming invasive and threatening the conservation of savanna ecosystems in Africa, for example, by displacing native vegetation or modifying the characteristics of invaded areas^12–14^. Therefore, research is needed to understand the factors driving plant invasions in these ecosystems.

Savannas are seasonal ecosystems characterized by the presence of grasses and patchily distributed trees and/or shrubs^10,15^. In these ecosystems, woody species facilitate their own establishment and growth, as well as that of other plant species. Due to their extensive root systems, trees and shrubs extract moisture and nutrients from their surroundings, accumulate litter and exude nutrients and other phytochemicals into surrounding soils. This leads to an accumulation of soil nutrients, increased humidity, and modification of microbial communities under their canopies^16^. The patchy distribution of trees in savannas can therefore lead to the creation of so-called “fertility islands”^17–19^, with favorable conditions for the establishment and growth of other native plants^20^. Therefore, it is feasible that fertility islands may also aid the establishment, growth, and impact of alien plants in savanna ecosystems. Moreover, dense populations of invasive plants may themselves create fertility islands by dramatically modifying soil biotic and/or abiotic conditions^21,22^. These fertility islands may have positive or negative impacts on the establishment and growth of other plants^23^.

Kruger National Park (KNP) is South Africa’s flagship protected area, covering about 2 million ha of savanna. Situated in north-eastern South Africa along the border of Mozambique, KNP is one of the largest protected areas in the world^24^. Plant invasions in KNP are some of the most studied in African savannas, especially from a protected areas perspective^12^. Although 363 alien plant (including non-invasive) species have been recorded in KNP, *Opuntia stricta* is one of only a few species currently considered a high-impact transformer species in the park (others include, for example, *Lantana camara* and *Parthenium hysterophorus*)^25,26^. Therefore, the invasion of *O. stricta* in KNP provides a good opportunity to assess potential drivers and impacts of plant invasions in African savannas^27^.

*Opuntia stricta* was first introduced to Skukuza village in KNP as an ornamental plant in 1953 and soon became invasive, covering 30,000 ha by 1998^28^. The invasion of *O. stricta* in KNP has been managed since 1985. An integrative management program, including chemical and biological control considerably reduced its density in the park (see Hoffmann et al.^29^). However, *O. stricta* is still expanding its range in both KNP^30^ and southern Africa^31^. In order to inform calls for management and understand its impacts, it is important to understand the biotic and/or abiotic factors driving, and responding to, *O. stricta* invasion.

Several hypotheses have been explored to explain the invasion success of *O. stricta* in KNP^9^. For example, Foxcroft et al.^32^ studied the influence of dispersal pathways, fire frequency, management history, soil type, substrate, vegetation type, and water regimes in the reproduction and survival of this species. However, besides the positive effect of the presence of the leguminous tree *Vachellia nilotica* subsp. *kraussiana* (hereafter *Vachellia nilotica*), none of the studied factors appeared to influence the invasion of *O. stricta* in the park^32^. Foxcroft and Rejmánek^33^ also tested the hypothesis that baboons (*Papio ursinus*) are major dispersal agents of *O. stricta* in KNP. *Opuntia stricta*, like many other *Opuntia* species, propagates both sexually, by producing large numbers of fleshy fruits and seeds^34^), and vegetatively, through plant fragments that root rapidly when in contact with soil^35^. Baboons eat the fruits and disperse both the seeds and fragments of *O. stricta* in KNP^28^. Foxcroft and Rejmánek^33^ therefore hypothesized that *O. stricta* plants would be more frequently found under trees structurally suitable for baboon roosting (e.g., *Spirostachys africana*) than under those that are not (e.g., *V. nilotica*), or on bare patches. However, they found *O. stricta* patches significantly more frequently under *V. nilotica* trees. Therefore, the landscape-scale factors driving *O. stricta* invasions in KNP remain unclear, and the potential influence of *V. nilotica* trees on the distribution of *O. stricta* requires further investigation.

The impact of *O. stricta* in KNP also deserves further study. Robertson et al.^36^ showed that, although the species does not affect native spider richness, diversity or community structure, it has an impact on native beetle assemblages in KNP. However, the impact of *O. stricta* on soil characteristics, soil bacterial communities, and the establishment of native plants, remains unknown.

Here, we compare soil characteristics (pH, humidity and nutrients), enzymatic activities, and the diversity and composition of bacterial communities between soils invaded by *O. stricta* and uninvaded soils, collected under the canopy of two native trees (*V. nilotica* and *S. africana*) and in open patches with no tree cover. With these data we aim to test whether (1) native trees and invasive *O. stricta* create fertility islands and, if so, whether (2) fertility islands influence the establishment of *O. stricta*. We also wanted to know (3) whether *O. stricta* modifies soils characteristics and soil bacterial communities in a different manner than native trees and (4) the effects of invasive *O. stricta* on soils affect the establishment of native trees. We hypothesise that native trees, but not *O. stricta*, will create fertility islands by increasing soil nutrient loads, humidity, soil enzyme activities and bacterial diversity and inducing changes in soil bacterial community composition. We also hypothesise that these fertility islands lead to enhanced performance of native trees and invasive *O. stricta*.

## Results

### Soil pH, humidity, nutrients and enzymatic activities

We found significant effects (p < 0.05) of invasion status (invaded, uninvaded) and/or tree cover (*V. nilotica*, *S. africana*, none) on soil humidity, organic matter, nitrogen, phosphorus, and phosphatase, β-glucosidase and urease activities (Table 1). Humidity, nutrients and phosphatase activity were higher under *V. nilotica* and *S. africana* canopies than under no canopy, and in invaded than in uninvaded areas. Moreover, in uninvaded areas, we generally found higher levels of nutrients and phosphatase activity under *V. nilotica* than under *S. africana* or in open patches with no trees. There were lower levels of ß-glucosidase and urease activities under the canopies of *V. nilotica* than under the canopies of *S. africana* or in open patches, regardless of invasion.

**Table 1:** Mean (± SE) of pH, humidity (%), organic matter (%), nitrogen (mg/Kg), phosphorus (mg/Kg), phosphatase activity (µmol/g h), β-glucosidase activity (µmol/g h) and urease activity (µmol/g h) in patches invaded and uninvaded by *O. stricta* under the canopies of *V. nilotica* and *S. africana* and in open patches with no tree cover.

Invasion	Uninvaded			Invaded		
Tree cover	*V. nilotica*	*S. africana*	None	*V. nilotica*	*S. africana*	None
pH	6.55 (0.05)	6.95 (0.28)	7.12 (0.15)	6.74 (0.03)	7.29 (0.22)	7.55 (0.51)
Humidity (%)	0.01^ab^ (0.00)	0.01^ab^ (0.00)	0.00^b^ (0.01)	0.03^a^ (0.00)	0.03^a^ (0.00)	0.05^a^ (0.02)
Organic matter (%)	1.03^ab^ (0.04)	0.60^bc^ (0.07)	0.31^c^ (0.03)	1.00^ab^ (0.20)	1.12^a^ (0.11)	0.64^abc^ (0.06)
Nitrogen (mg/kg)	24.00^a^ (2.89)	17.33^b^ (1.76)	8.33^c^ (1.45)	26.33^a^ (3.84)	31.33^a^ (6.12)	20.33^ab^ (5.84)
Phosphorus (mg/kg)	21.33^a^ (1.76)	13.67^ab^ (2.67)	4.33^b^ (2.03)	12.67^ab^ (3.84)	10.50^ab^ (3.51)	16.00^ab^ (4.36)
Phosphatase (µmol/g h)	2.93e−02^a^ (2.97e−04)	1.93e−02^b^ (3.19e−03)	6.43e−03^c^ (1.53e−03)	2.10e−02^ab^ (3.20e−03)	2.85e−02^a^ (5.00e−04)	2.60e−02^a^ (1.71e−03)
ß-glucosidase (µmol/g h)	5.17e−03^bc^ (1.52e−03)	1.34e−02^a^ (1.59e−03)	9.73e−03^ab^ (1.23e−03)	3.88e−04^c^ (3.88e−04)	1.22e−02^a^ (1.63e−03)	1.18e−02^a^ (1.20e−03)
Urease (µmol/g h)	3.27e−05^c^ (8.15e−06)	5.15e−04^a^ (7.83e−05)	1.57e−04^bc^ (7.00e−06)	3.37e−05^c^ (1.29e−05)	5.60e−04^a^ (1.94e−05)	1.92e−04^b^ (1.64e−05)

Letters indicate significant differences between soils (P < 0.05).

### Soil bacterial community diversity and composition

We obtained 884,540 high-quality sequence reads after data cleaning, which resulted in 18,717 OTUs (97% similarity cut-off) representing 325 genera, 141 families, 75 orders, 40 classes, and 17 phyla. The following numbers of sequences remained unclassified: Genus level—43.9% of all sequences (388,210); Family level—28% (247,259); Order level—14.4% (127,477); Class level—3.7% (32,356); and Phylum level—3.2% (27,891) (Supplementary material). The most abundant phyla were Actinobacteria (50.8%), followed by Proteobacteria (32.9%) and Firmicutes (6.6%). At class level Actinobacteria (50.7%) were most abundant, followed by Alphaproteobacteria (27.7%), Bacilli (6.5%), Betaproteobacteria (2.9%), and Deltaproteobacteria (2.2%).

**Figure 1:**
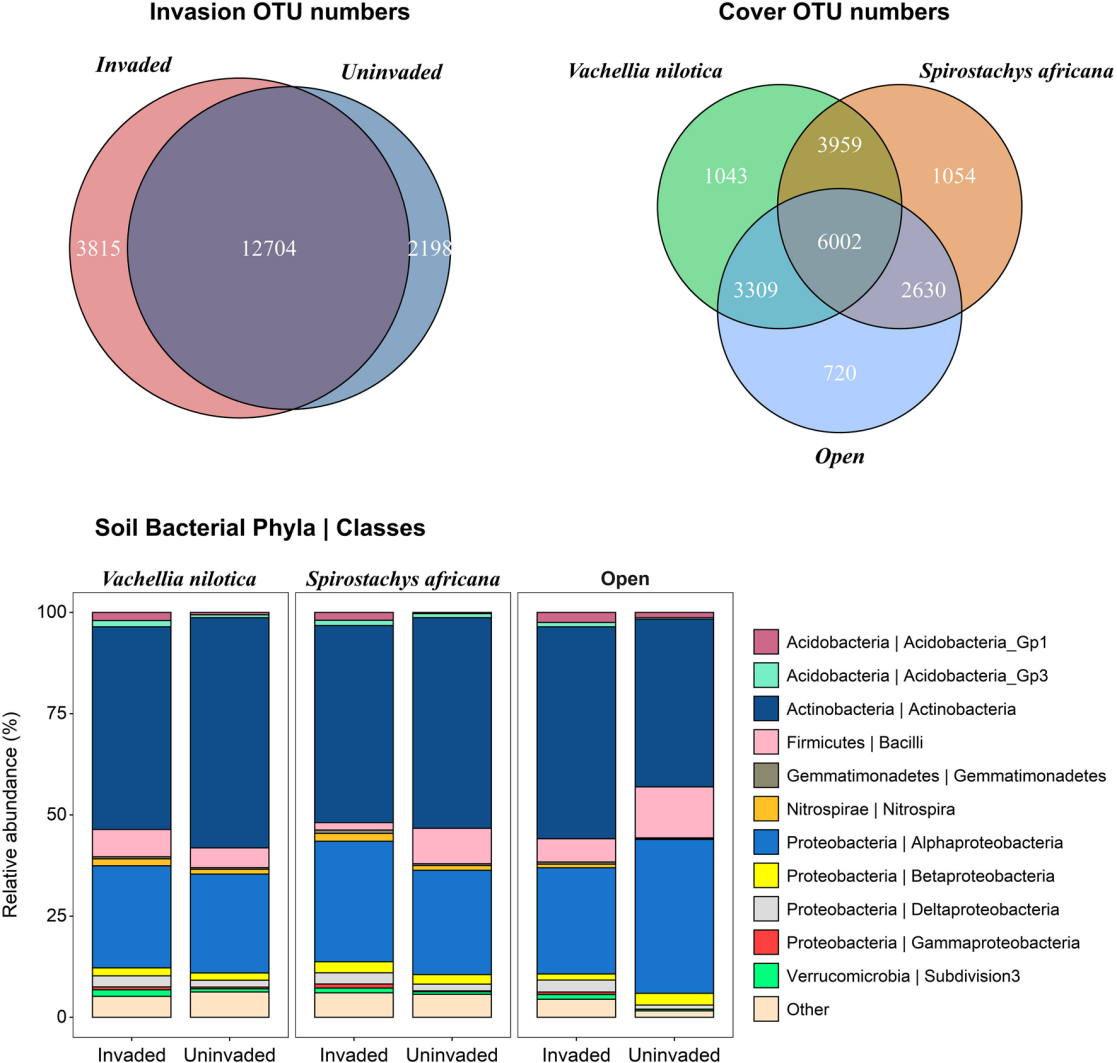
Distribution of OTUs (operational taxonomic units) between invasion (invaded and uninvaded; top left) and tree cover (*V. nilotica*, *S. africana*, none; top right) categories, together with relative abundances of soil bacterial taxa (Class | Phylum). Class-level relative abundances were calculated using the number of sequences for each taxon as a percentage of the total sequences for each invasion/cover combination. The “Other” category includes taxa that were unclassified at Class level and classes representing less than 0.5% of the total number of sequences.

A total of 12 704 OTUs (861 232 reads, 97.4% of total read count) were shared between invaded and uninvaded soils (Fig. 1), whereas 3815 OTUs (16 172 reads, 1.8% of total read count) were unique to invaded soils and 2198 OTUs (7 136 reads, 0.8% of total sequence read count) to uninvaded soils. For tree cover, a core total of 6002 OTUs was shared by all soils (Fig. 1), and also made up the bulk of the sequence reads (834,286 reads, 94.3% of total read count). However, there were OTUs that were unique to each tree cover type: 720 OTUs (1839 reads, 0.21% of total read count) were unique to open areas, 1043 OTUs (2912 reads, 0.33% of total read count) were unique to *V. nilotica* soils, and 1054 OTUs (3439 reads, 0.39% of total read count) were unique to *S. africana* soils.

**Figure 2:**
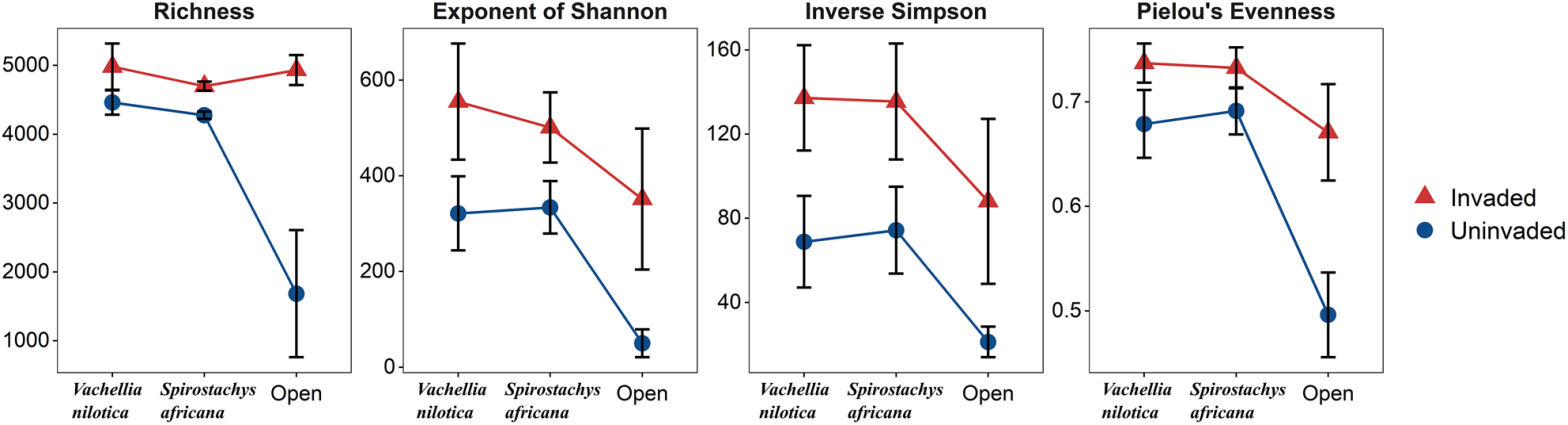
Diversity metrics for soil bacterial communities associated with *O. stricta* invasion (i.e. invaded vs. uninvaded) and tree cover (i.e. *V. nilotica*, *S. africana*, none) categories. Error bars represent the standard deviation of the mean.

**Figure 3:**
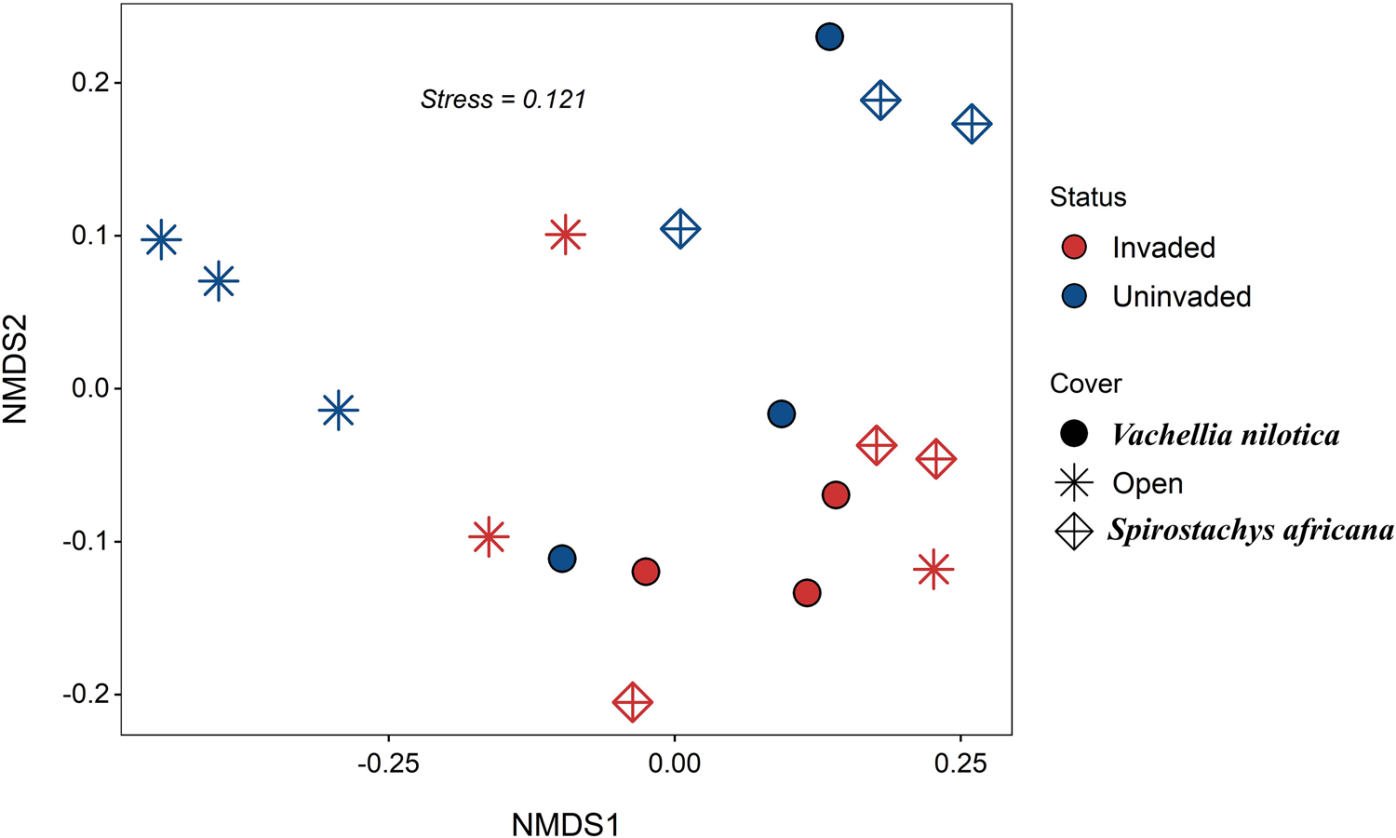
Non-metric Multidimensional Scaling (NMDS) plot for soil bacterial communities for various invasion (Invaded, Uninvaded) and tree cover (*V. nilotica*, *S. africana*, none) categories. PERMANOVA results confirmed the significant effects of both cover and invasion status on bacterial community composition. The relatively low stress coefficient indicates good ordination with no risk of drawing false inferences^89^.

Two-way ANOVAs indicated that the presence of native trees increased the diversity of bacterial communities, especially in uninvaded soils. Furthermore, all diversity metrics were significantly affected by invasion status, being higher in invaded than in uninvaded soils (Table 2, Fig. 2). The presence of *V. nilotica*, *S. africana*, and *O. stricta* also significantly altered soil bacterial community structure and composition based on Horn distances (Table 3, Fig. 3).

**Table 2:** ANOVA results on the differences in diversity of microbial communities between patches invaded and uninvaded by *O. stricta* under the canopies of *V. nilotica* and *S. africana* and in open patches with no tree cover.

Diversity	Factor	Df	Mean sq	F	p
Richness	Invasion	1	8,778,050	16.7	0.002**
	Tree cover	2	3,442,078	6.6	0.012*
	Invasion × tree cover	2	3,855,228	7.3	0.008**
Exponent of Shannon	Invasion	1	246,208	9.5	0.009**
	Tree cover	2	103,953	4.0	0.046*
	Invasion × tree cover	2	6743	0.3	0.775
Inverse Simpson	Invasion	1	19,264.1	9.9	0.008**
	Tree cover	2	4868.2	2.5	0.123
	Invasion × tree cover	2	21.8	0.0	0.989
Pielou’s evenness	Invasion	1	0.0375	12.4	0.004**
	Tree cover	2	0.0320	10.6	0.002**
	Invasion × tree cover	2	0.0079	2.6	0.114

Significance indicated in bold as follows: *p < 0.05; **p < 0.01.

**Table 3:** PERMANOVA results. Significance indicated in bold as follows: *p < 0.05; **p < 0.01.

Factor	Df	Sum of squares	F	p
Tree cover	2	0.672	2.53	0.004**
Invasion	1	0.371	2.79	0.01*
Invasion × tree cover	2	0.404	1.52	0.0916
Residuals	12	1.595

Analysis of multivariate homogeneity of group dispersions indicated that uninvaded soils were significantly more over dispersed (BETADISPER F_1,16_ = 8.76, p = 0.009), suggesting that invasive *O. stricta* plants have a homogenizing effect on soil bacterial communities, i.e. areas invaded by *O. stricta* had more similar bacterial communities than uninvaded areas. The effect of tree cover did not, however, have a significant homogenizing effect (BETADISPER F_2,15_ = 0.15, p = 0.865).

**Figure 4:**
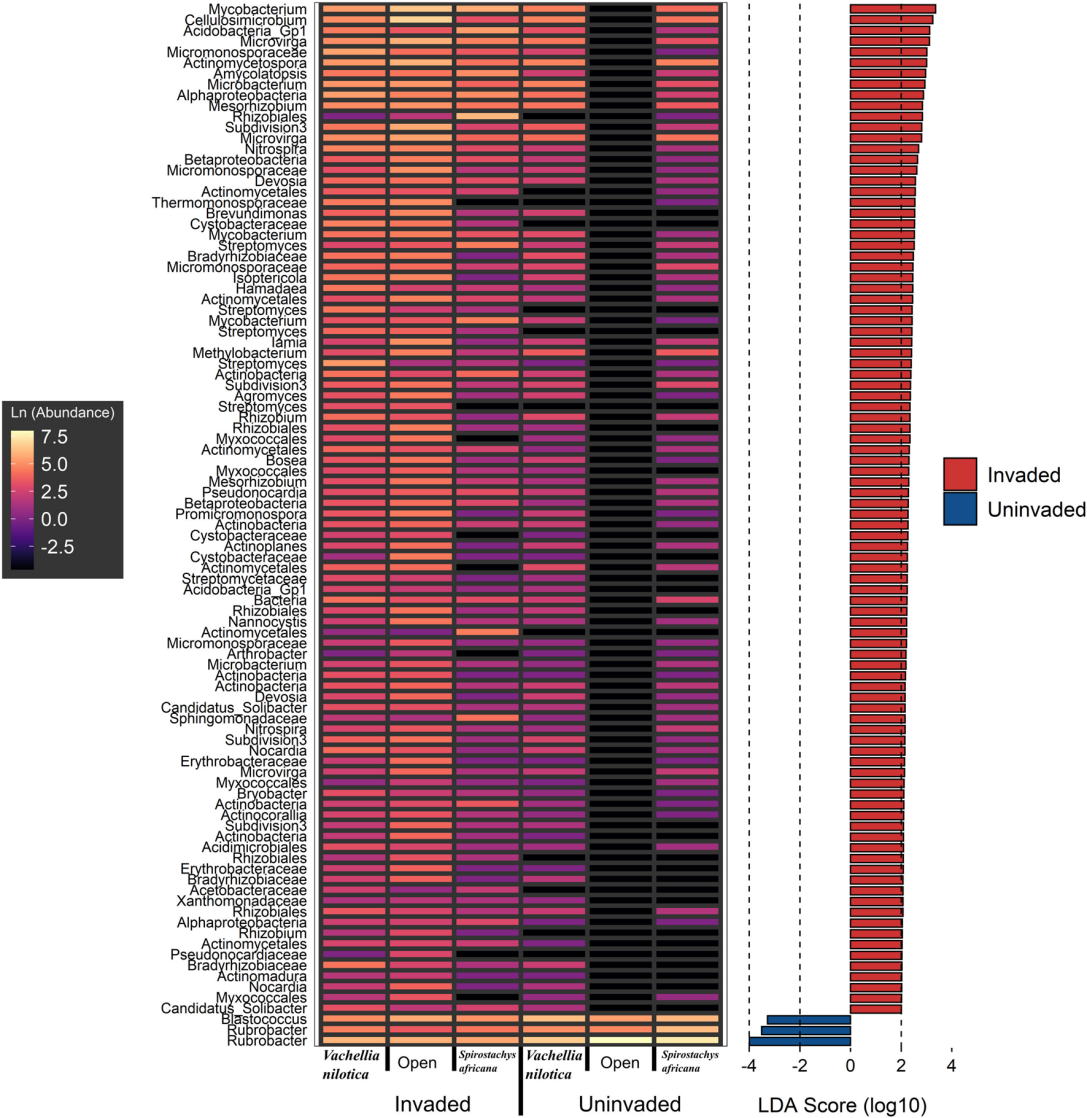
Bacterial taxa that are significantly differentially abundant between status (invaded, uninvaded) and tree cover (*V. nilotica*, *S. africana*, none). Shown on the left is a heatmap of the taxa with Ln(Abundance) values, and on the right the LEfSe LDA scores for each taxon^88^. Only significant LDA scores are shown. The red and blue colours associated with the LDA scores indicate to which categories the taxa were associated with. Note that a small constant (0.01) was added to heatmap abundance values to avoid the undefined natural logarithm of 0.

Our linear discriminant analysis effect size (LEfSe) highlighted numerous OTUs (n = 94) that were significantly more abundant in invaded areas, irrespective of cover type (Fig. 4). This indicates that *O. stricta* invasion leads to a significant increase in the abundance of these OTUs. On the other hand, only three OTUs (two belonging to *Rubrobacter* and one to *Blastococcus*) were significantly more abundant in uninvaded soils.

### Germination

The percentage and speed of germination of *O. stricta* were generally higher in soils collected under *V. nilotica* canopies and in soils from uninvaded areas (Table 4). The percentage and speed of germination of *V. nilotica* were significantly lower under uninvaded soils collected from underneath its own canopy. We found no significant differences in the percentage and speed of germination of *S. africana*.

**Table 4:** Mean (± SE) of total germination (Gt) and speed of germination (AS) of *O. stricta*, *V. nilotica* and *S. africana* in patches invaded and uninvaded by *O. stricta* under the canopies of *V. nilotica* and *S. africana* trees and in open patches with no tree cover.

Species	Invasion	Uninvaded			Invaded		
	Tree cover	*V. nilotica*	*S. africana*	None	*V. nilotica*	*S. africana*	None
*O. stricta*	Gt	0.467^a^ (0.038)	0.388^b^ (0.040)	0.333^bc^ (0.058)	0.367^b^ (0.033)	0.192^c^ (0.028)	0.283^c^ (0.044)
	AS	0.395^a^ (0.091)	0.266^ab^ (0.066)	0.371^ab^ (0.119)	0.138^b^ (0.066)	0.054^b^ (0.019)	0.351^ab^ (0.072)
*V. nilotica*	Gt	0.107^b^ (0.012)	–	0.273^a^ (0.029)	0.253^a^ (0.025)	–	0.300^a^ (0.043)
	AS	0.140^b^ (0.030)	–	0.346^a^ (0.020)	0.212^ab^ (0.041)	–	0.279^ab^ (0.051)
*S. africana*	Gt	–	0.222 (0.080)	0.178 (0.022)	–	0.311 (0.022)	0.267 (0.102)
	AS	–	0.378 (0.114)	0.239 (0.054)	–	0.496 (0.048)	0.277 (0.130)

Letters indicate significate differences between soils (P < 0.05).

## Discussion

Woody species in savannas accumulate moisture and soil nutrients, and harbor unique bacterial communities under their canopies, creating “fertility islands”^16^. Accordingly, and in support of our hypothesis, our results show higher soil humidity, nutrient contents, and phosphatase activity under the canopies of *V. nilotica* and *S. africana* trees in KNP compared to open patches with no tree cover. Interestingly, the same soil physicochemical properties were also elevated as a result of *O. stricta* invasion. These results are surprising, since previous studies have shown that cacti in arid ecosystems generally provide little shade and limited amounts of aboveground litter, and therefore, that soils beneath them have similar humidity and nutrient levels than those in bare patches^37,38^.

Differences in morphology, physiology, or root symbiosis of woody species can cause differences in the characteristics of the fertility islands they create^39^. Accordingly, we found higher concentrations of soil nutrients and phosphatase activity under *V. nilotica* canopies than under *S. africana* canopies. This can be explained by the fact that acacias have rapid growth rates and the capacity to fix atmospheric nitrogen via rhizobium symbioses, and legumes in general have been repeatedly shown to induce changes in soil nutrient levels and cycles^40–44^. Moreover, *V. nilotica* has previously been found to increase soil nutrient concentrations due to high above- and belowground organic matter input^45^. In particular, the presence of *V. nilotica* trees in our uninvaded areas caused a significant increase in soil nitrogen content, which is a particularly limiting macronutrient in the study area^10^.

It has also been suggested that, due to leaf litter input, *V. nilotica* trees increase nutrient cycling^45^. However, we found significantly lower levels of β-glucosidase and urease activities under *V. nilotica* canopies. These results are unexpected, and more research is needed to unravel the mechanisms underlying these observations.

In agreement with our findings, previous studies have reported the phyla *Actinobacteria* and *Firmicutes* as abundant bacterial groups in semi-arid savannas^46^. *Actinobacteria* taxa are generally drought and heat resistant^47^, while many *Firmicutes* are spore-forming, an adaptation to harsh and unpredictable environmental conditions^48^.

Our results showed that the presence of *V. nilotica*, *S. africana*, and *O. stricta* altered soil bacterial composition and increased relative bacterial abundance and diversity compared to open patches with no tree cover. This likely reflects the increases in soil humidity and nutrient contents we observed under these plants^49–51^. However, increases in bacterial abundance and diversity were more pronounced in *O. stricta*-invaded soils and *O. stricta* invasion had a significant homogenizing effect on soil bacterial communities. These results suggest that fertility islands created by *O. stricta* might have stronger effects on the diversity and composition of bacterial communities of savanna soils than those created by the native woody species.

Seeds have various mechanisms to detect suitable conditions for establishment and to adjust their timing of germination accordingly^52,53^. Our results suggest that the germination of *O. stricta* in the study area might be facilitated by the presence of *V. nilotica* trees (also see^32,33^). Such enhanced germination kinetics may be the result of the higher soil nutrient levels present under the canopy of these trees compared with those under the canopy of *S. africana* or in open patches with no tree cover, which might trigger the germination of *O. stricta* seeds.

Timing of germination can also be adjusted as a response to chemicals released by conspecific or other plant species^54^. Such mechanisms can help plants to avoid intra- or inter-specific competition or to detect the presence of facilitating or nursing species, maximizing establishment potential^55^. Accordingly, while *V. *nilotica and *S. africana* trees appear to germinate equally well in invaded and uninvaded soils, we found *O. stricta* to have reduced germination performance (lower and slower) in invaded soils. These results suggest the potential release of phytochemicals by *O. stricta*, which may retard its own germination, i.e. the creation of negative soil-feedbacks, aiming to avoid potential intraspecific competition. Similarly, we also found *V. nilotica* to have lower germination performance in uninvaded soils collected under its own canopy. These results are not surprising, since previous studies have found different species in the genus *Acacia* (in which *V. nilotica* previously resided) to adjust their timing of germination as a response to chemically-induced signals released by adult plants^56^. Moreover, *V. nilotica* is known to produce several chemicals, including tannins, flavonoids, and phenolic acids, capable of stimulating or inhibiting seed germination^57^.

Overall, our results showed that native tree cover and invasive species can create fertility islands in savanna ecosystems, causing changes to the abiotic and biotic conditions of soils. This suggests that the presence of any tree or shrub in savanna ecosystems, regardless of origin (i.e. native or alien) or growth from (e.g. woody tree or succulent shrub), can result in the formation of fertility islands, which usually create favourable conditions for the establishment and growth of other plants^20^. We also found the presence of *V. nilotica* trees to be linked to higher increases in soil nutrient contents than the presence of *S. africana* trees and that these increases might favour the germination of invasive *O. stricta* seeds. These results provide a mechanistic basis to previous studies suggesting that establishment of *O. stricta* in KNP might be facilitated by the microenvironment created by *V. nilotica* trees^32^. Finally, invasive *O. stricta* did not affect the germination of native trees, but affected soil bacterial communities more strongly than native trees. Since the eradication of *O. stricta* in KNP is no longer feasible^30^, the consequences of these effects deserve further investigation.

## Methods

All the local and national guidelines were followed in this study. A contract between the researchers and SANParks (permit NOVOA1292) provided all formal permission needed to conduct the research (which fulfils the NEMBA: Protected Areas Act).

### Study site

**Figure 5:**
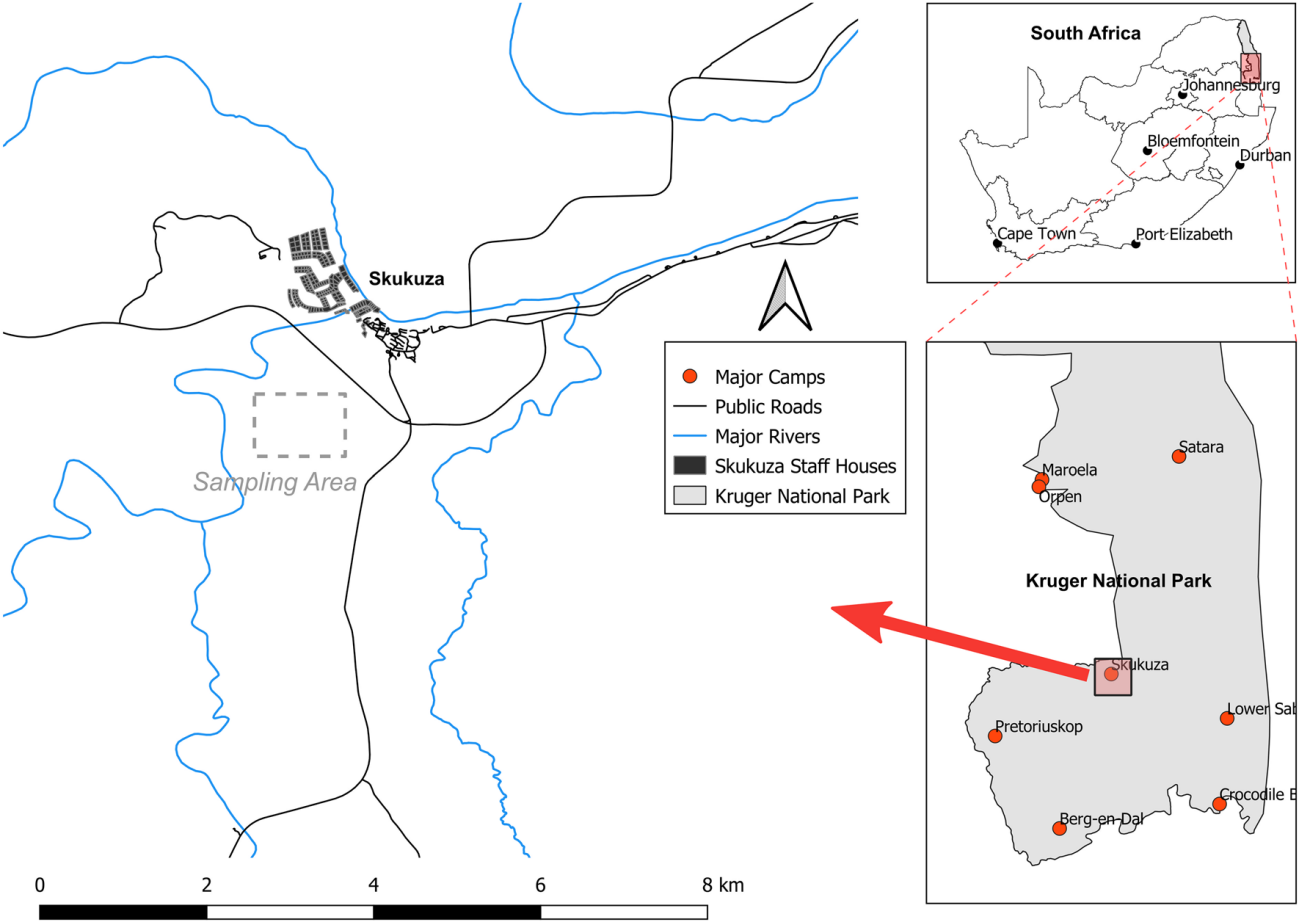
The study site is situated approximately 1 km south-west of the Skukuza Rest Camp in the south of Kruger National Park, South Africa.

**Figure 6:**
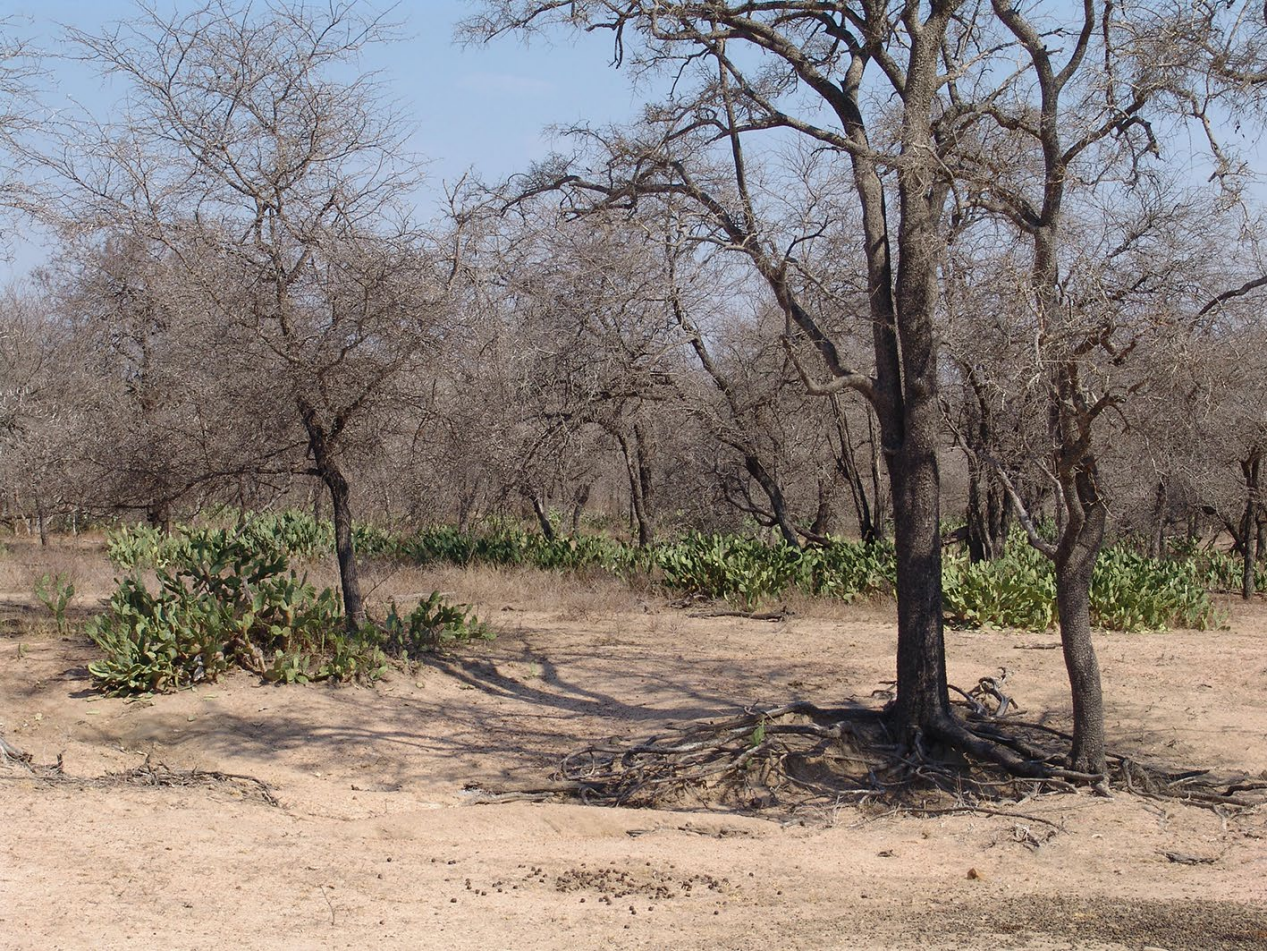
*Opuntia stricta* growing under de canopy of *Vachellia nilotica* (background), while no *O. stricta* plants are visible under the canopy of *Spirostachys africana* (front right) or in open patches with no tree cover.

Our study site was located in the south of KNP (Fig. 5), approximately 1 km south-west of Skukuza Rest Camp (− 25.0049, 31.5852), in a typical savanna vegetation with sparse grass cover, open patches with no tree cover, and a patchy distribution of trees (Fig. 6). Two woody species, *V. nilotica* and *S. africana*, make up more than 90% of all trees in the study site, and their individuals are mixed in the landscape. The study site has low grass cover, minimising herbivory by native ungulates and due to the open patchy landscape fire in the immediate area is minimal. Moreover, the study site presents granitic geological substrate and thus has nutrient-poor soils^58^, suggesting a high importance of fertility islands for the establishment of plants in the area.

### Study species

*Vachellia nilotica* (previously *Acacia nilotica*^59^), is a semi-deciduous tree in the Fabaceae (legume) family. It can grow up to 10 m in height and its branches are armed with straight, paired spines. *Vachellia nilotica* is widely distributed in southern Africa, from Tanzania to South Africa^60^. *Spirostachys africana* is a spineless deciduous tree from the Euphorbiaceae (spurge) family. It can grow up to 18 m in height, although it usually only reaches about 10 m. *Spirostachys africana* is also widely distributed in southern Africa^61^.

*Opuntia stricta* is a perennial, succulent, shrubby plant in the Cactaceae (cactus) family and is native to Cuba, Mexico, and the USA^34^. Commonly introduced around the world as an ornamental plant, *O. stricta* is currently considered invasive in 21 countries^31^. In its invasive ranges, *O. stricta* causes multiple ecological and socioeconomic impacts. It reduces agricultural production and biodiversity, causes loss of grazing potential, transforms habitats, and causes injuries to animals and people via its spines^62^.

### Soil and seed collection

We collected samples of *O. stricta*-invaded and uninvaded soils under the canopies of *V. nilotica* and *S. africana* and in open patches with no tree cover. The identification of the plant species was undertaken by AN, LCF and JJLR. No voucher specimens were collected or deposited in a publicly available herbarium. In each of these six soil types, we randomly established five plots of 0.5 × 0.5 m. We did not establish more than one plot under the canopy of a single tree. In each plot, we took five subsamples from the top 10 cm of soil using a shovel. The subsamples within each plot were then sieved through a 2.0 mm mesh and homogenized into a single sample, resulting in 30 samples in total (2 invasion status [invaded, uninvaded] × 3 tree covers [*V. nilotica*, *S. africana*, none] × 5 plots × 1 sample per plot). All equipment was cleaned and sterilized with ethanol between collections to avoid cross contamination. For each soil sample we measured (1) pH, humidity, and nutrients (2) soil enzymatic activities, and (3) germination of *O. stricta*, *V. nilotica* and *S. africana*. Moreover, we randomly selected 3 samples per treatment, resulting in 18 samples in total (2 invasion status [invaded, uninvaded] × 3 tree covers [*V. nilotica*, *S. africana*, none] × 3 plots × 1 sample per plot), to measure the diversity and composition of soil bacterial communities. Soils used for pH, humidity, nutrients, and germination analyses were kept at room temperature. Soils used for enzymatic activity analysis were refrigerated at 4 °C and analysed within 3 days of collection. Soils used for DNA extraction (i.e. for next-generation sequencing analyses of bacterial communities) were kept on ice during transport and stored at −80 °C as soon as was possible.

*Opuntia stricta, V. nilotica* and *S. africana* seeds were also collected in the study area and stored in the dark at 4 °C. *O. stricta* is declared as invasive in the park, and therefore no permission was needed to collect its seeds. Seeds of the native trees were collected through the Skukuza plant nursery, which has permission to collect and store such seeds, as well as germinate and sell them.

### Soil pH, humidity, nutrients and enzymatic activities analysis

Soil pH was determined by dilution with water (1:2.5; soil: distilled water)^63^, using a CRISON GLP 22 + pH & Ion-Meter. To determine soil humidity, we first weighed the fresh soil samples, and then dried (70 °C for 48 h) and reweighed them, calculating soil humidity as: (Fresh soil weight—Dry soil weight)/(Fresh soil weight) × 100. Soil nutrients (nitrogen, organic matter, and phosphorous) were measured by Labserve Laboratories (Nelspruit, South Africa).

We also analysed three enzymes that play key roles in soil nutrient cycling: β-glucosidase (E.C. 3.2.1.21), involved in carbon metabolism through the release of glucose from cellulose; urease (E.C 3.5.1.5), involved in the release of nitrogen by degrading urea to ammonium; and phosphatase (E.C. 3.1.3.1), involved in the release of phosphate from organic matter by hydrolyzing phosphate ester bonds^52^. We used the methods described by Tabatabai and Bremner^64^, Kandeler and Gerber^65^ and Allison and Vitousek^66^, for the β-glucosidase, urease, and phosphatase assays, respectively. Following the recommendation of German et al.^67^, we conducted the enzyme assays at environmental pH conditions and within 48 h of soil collection.

### DNA extraction and next generation sequencing

We extracted whole genomic DNA from 0.25 g of soil using the PowerSoil^®^ DNA extraction kit (MO BIO laboratories Inc., Carlsbad, CA, USA), following the manufacturer's protocol. We assessed DNA quality using the NanoDrop ND-1000 UV–Vis Spectrophotometer (Nanodrop Technologies, Wilmington, DE, USA). Part of the 16S rRNA gene (consisting of nine hypervariable regions: V1–V9) was targeted for amplification, since it is frequently used for the identification of bacterial taxa^68,69^. The more variable regions are useful for genus- or species-level identifications^70^. We targeted the V5-V7 hypervariable regions using primers 799F (5'-AAC MGG ATT AGA TAC CCK G-3') and 1391R (5'-GAC GGG CGG TGW GTR CA-3'). These primers are known for low non-specificity, and can accurately and reproducibly differentiate species^71–73^. Amplification was done with sample-specific barcodes in the forward primer, using a 30 cycle PCR and the HotStarTaq Plus Master Mix Kit (Qiagen, Valencia, CA, USA) under the following PCR conditions: 94 °C for 3 min, followed by 30 cycles of 94 °C for 30 s, 53 °C for 40 s and 72 °C for 1 min, followed by a final elongation at 72 °C for 5 min. We checked the PCR products on a 2% agarose gel to determine the success of amplification and the relative intensity of bands. Multiple PCR samples were then pooled together in equal proportions based on their molecular weight and DNA concentrations. Pooled samples were purified using calibrated Ampure XP beads (Agencourt Bioscience Corporation, Beverly, MA, USA) and were used to prepare DNA libraries following the Illumina TruSeq DNA library preparation protocol. We sequenced the samples using the Molecular Research LP next generation sequencing service (https://www.mrdnalab.com, Shallowater, TX, USA) on an Illumina MiSeq instrument (Illumina, San Diego, CA, USA) following the manufacturer’s guidelines.

### Bioinformatics and taxonomic identification

We processed all raw MiSeq DNA sequence data following standard procedures as described in Schloss et al.^74^ using the mothur version 1.37.1^75^. First, we removed low quality sequences and optimized the sequence lengths (to between 385 and 395 bp). We then aligned unique sequences to the SILVA-ARB reference database (release 123) to the same region of the 16S rRNA gene we sequenced and removed those columns that contained gaps only. Furthermore, independent of a reference database, we removed all the chimeric sequences using the uchime algorithm^76^ and the template as self, i.e. de novo removal. Subsequently, we clustered sequences into operational taxonomic units (OTUs) at the 97% DNA sequence similarity level. Representative sequences for OTUs were chosen as those that were most abundant in each cluster. We determined the taxonomic identity of each OTU with the ribosomal database project (RDP) Classifier^77^, and all sequences classified as chloroplast, mitochondria, and Archaea were removed. In order to standardize the number of reads across all samples, we subsampled (i.e. rarefied) equivalent reads from each sample. Rarefaction is believed to increase the false discovery rate^78^, but this is not true^79^ and instead, rarefying can lower sensitivity (false negatives) as a result of data discarding. Thus, recommendations are to rarefy to the highest depth possible^80^, which is what we did. Rarefaction is still considered a useful normalization technique, especially for uneven library sizes between groups, like here, and results in a higher PERMANOVA *R*^2^ for studied biological effects. However, it should still be noted that some OTUs can potentially be lost during rarefaction.

### Germination experiments

Seeds of all three species were surface-sterilized for 5 min in 1% sodium hypochlorite, rinsed 3 times in distilled water, and dried at room temperature prior to the experiment to avoid fungal contamination. Thirty randomly selected seeds of *O. stricta* were sowed in petri dishes filled with 2 g of soil collected in invaded and uninvaded areas under the canopies of *V. nilotica* and *S. africana* and in open patches with no tree cover. Each treatment was replicated four times (*n* = 2 invasion scenarios [invaded, uninvaded] × 3 tree covers [*V. nilotica*, *S. africana*, none] × 4 replicates = 24 petri dishes). The same process was followed to test the germination of *V. nilotica* and *S. africana* in soils collected in invaded and uninvaded areas under their own canopy and in open patches with no tree cover (*n* = 2 species [*V. nilotica*, *S. africana*] × 2 invasion scenarios [invaded, uninvaded] × 2 tree covers [under its own canopy, none] × 4 replicates = 32 petri dishes). Petri dishes were moistened with 2 mL distilled water every 2 days and incubated in a germination chamber with periods of 12/12 h of light/dark and 25/15 °C day/night temperatures. Seeds were considered germinated when the seed coat was broken and the radicle visible (Posmyk et al. 2009). The number of germinated seeds was recorded every 2 days until no new seeds germinated. Total accumulated germination was calculated as Gt = [N_T_ × 100]/N, where N_T_ is the total germinated seeds in each treatment and N is the total number of seeds used in the treatment. Speed of accumulated germination was calculated as AS = [N_1_/1 + N_2_/2 + N_3_/3 … + N_n_/n], where N_1_, N_2_, N_3_, N_n_ is the cumulative number of seeds which germinated at a 2-day interval 1, 2, 3, … N^81^.

### Statistical analyses

All statistical analyses were conducted in the R statistical environment (version 3.5.1) (R Core Team 2017), unless otherwise specified. Where no package is indicated, functions used were from the vegan package version 2.3-3^82^.

OTU accumulation curves were generated with the function specaccum to determine whether sampling was adequate to detect all OTUs present. Four diversity metrics were calculated from the sample x OTU matrix: OTU richness, the exponent of Shannon diversity, inverse Simpson diversity, and Pielou's evenness (OTU abundance equality)^83,84^. The exponent of Shannon and Inverse Simpson diversities were chosen since these metrics represent true diversities (i.e. "effective species"), unlike other diversity indices/entropies^83,84^. We calculated these various metrics with the function renyi. In order to investigate the influence of invasion (invaded vs. uninvaded) and tree cover (*V. nilotica*, *S. africana*, none) on the various diversity metrics, we performed two-way ANOVAs, including interaction effects. Significant differences between means were assessed using Tukey HSD post hoc tests.

For visualizing soil bacterial community composition, we performed Non-Metric Multidimensional Scaling (NMDS) using function metaMDS based on Horn similarity values^85^ for the 97% OTU table. Horn similarity values were calculated with the function sim.table in the vegetarian package^86^. To test for significant differences in soil bacterial community composition between different soils, we performed a Permutation Multivariate Analysis of Variance (PERMANOVA)^87^ with 9999 permutations using the function adonis.

We were interested whether the presence of native trees or invasive *O. stricta* homogenized soil bacterial communities. We tested this with the betadisper function (using 9999 permutations), which calculates differences in multivariate homogeneity of group variances between tree covers, and invaded and uninvaded soils.

To identify shifts in the abundance of OTUs due to the presence of native trees or the invasive *O. stricta*, we performed a linear discriminant analysis (LDA) effect size (i.e. LEfSe)^88^ using mothur^75^ at OTU level. LEfSe taxa are those that have shifted in abundance (identified using an alpha value of 0.05 and LDA score of > 2). Finally, we investigated which OTUs were broadly present in all soils as these are presumably not affected by invasion or tree cover.

Finally, in order to investigate the influence of invasion (invaded vs. uninvaded) and tree cover (*V. nilotica*, *S. africana*, none) on the soil pH, humidity, nutrients and enzymatic activities datasets and the germination indices, we performed two-way ANOVAs, including interaction effects. Significant differences between means were assessed using Tukey HSD post hoc tests.

## Supplementary Information

Supplementary Table S1.

Supplementary Information.

## Data Availability

All data generated or analysed during this study are included in this published article (and its Supplementary Information files).
