# Supplementary material for: The invasive cactus Opuntia stricta creates fertility islands in African savannas and benefits from those created by native trees
Source: Sci Rep. 2021 Oct 21;11:20748. doi: 10.1038/s41598-021-99857-x (PMC8531129; doi:10.1038/s41598-021-99857-x)
Supplement: Supplementary file 1 — Supplementary Table S1. [file 41598_2021_99857_MOESM1_ESM.docx]

## Supporting Information

Article title: Invasive plants create fertility islands in African savannas and benefit from those created by native trees

The following Supporting Information is available for this article:

**Table S1** BETADISPER results.

| Factor | Df | Mean Sq | F | p |
| --- | --- | --- | --- | --- |
| Invasion | 1 | 0.036 | 8.76 | **0.009*** |
| Residuals | 16 | 0.004 |  |  |
|  |  |  |  |  |
| Tree cover | 2 | 0.001 | 0.146 | 0.865 |
| Residuals | 15 | 0.009 |  |  |

Significance indicated in bold as follows: * p < 0.05

Dataset S1. OTU Table

Dataset S2. Taxonomy of the identify OTUs
